# Stage, grade and morphology of tumours of the colon and rectum recorded in the Oxford Cancer Registry, 1995–2003

**DOI:** 10.1038/sj.bjc.6603499

**Published:** 2006-11-28

**Authors:** J Green, J Watson, M Roche, V Beral, J Patnick

**Affiliations:** 1 Nuffield Department of Clinical Medicine, Cancer Research UK Epidemiology Unit, University of Oxford, Richard Doll Building, Roosevelt Drive, Oxford OX3 7LF, UK; 2 Oxford Cancer Intelligence Unit, 4150 Chancellor Court, Oxford Business Park South, Oxford OX4 2GX, UK; 3 NHS Cancer Screening Programmes, Fulwood House, Old Fulwood Road, Sheffield S10 3TH, UK; 4 NHS Cancer Screening Programmes Research Unit, Richard Doll Building, Roosevelt Drive, Oxford OX3 7LF, UK

**Keywords:** colorectal cancer, screening, cancer registration

## Abstract

Data on stage, grade and morphology of 12 761 colorectal cancers registered between 1995 and 2003 by Oxford Cancer Registry are reviewed. Dukes stage is recorded for 81% of colon cancers and for 69% of rectal cancers. Incomplete registry data and changing recording practices may affect future evaluation of bowel cancer screening.

The UK NHS Bowel Cancer Screening Programme (http://www.cancerscreening.nhs.uk/bowel) was introduced this year. In order to evaluate its impact on incidence and mortality for colorectal cancer, reliable data will be needed on trends in registrations for cancers of the colon and rectum, and in particular on tumour stage distribution, before and after the introduction of screening. Tumour stage is the main determinant of survival from colorectal cancer, and is expected to change substantially with screening. Data on stage and grade have formed part of the National Minimum Dataset for colorectal cancer collected by UK Cancer Registries since 1995, but are not included in published national statistics (Hayne *et al*, 2001; Quinn *et al*, 2001). We describe data on colorectal cancer held by the Oxford Cancer Intelligence Unit for the years 1995–2003.

## MATERIALS AND METHODS

The Oxford Cancer Registry collects data on ∼1400 incident cases of colorectal cancer each year in Berkshire, Buckinghamshire, Northamptonshire and Oxfordshire (a population of ∼2.8 million). Since the early 1990s data have been obtained electronically directly from hospital pathology, oncology and patient administration records and from supplementary sources such as private hospitals and the Office for National Statistics. For invasive cancers of the colon (ICD10 C18) and of the rectum and related sites (ICD10 C19–21) registered in 1995–2003, the following variables were examined: year of diagnosis, age at diagnosis (in years), sex, Dukes stage (A, B, C, D or not staged/not stageable), grade (well, moderately, poorly or un-differentiated or not known) and morphology (using a histological classification based on that of WHO for tumours of the colon and rectum; Hamilton and Aaltonen, 2000). Age-standardised registration rates were calculated by direct standardisation using the population of the catchment area in 2000 as reference.

## RESULTS

**Figure 1:**
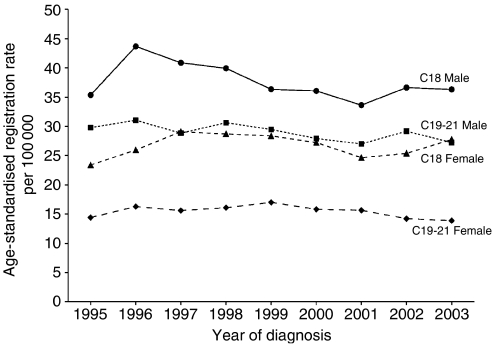
Oxford Cancer Registry (1995–2003): age-standardised registration rates for cancers of the colon (C18) and of the rectum and related sites (C19–21), by sex.

**Figure 2:**
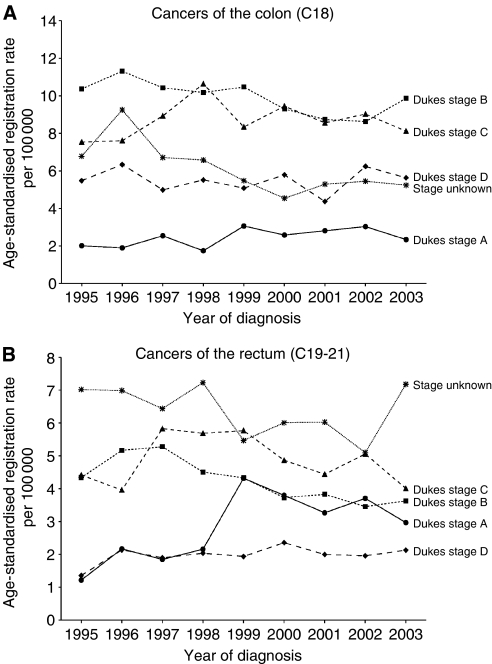
Oxford Cancer Registry (1995–2003): age-standardised registration rates for cancers of the colon (C18) (**A**) and of the rectum and related sites (C19–21) (**B**), by Dukes stage.

Between 1995 and 2003, a total of 12 761 incident invasive colorectal cancers, 7865 of the colon and 4896 of the rectum and related sites (including 836 cancers of the rectosigmoid junction or of colon with rectum, C19; 3778 of rectum, C20, and 282 of anus and anal canal, C21), were registered. Characteristics of these cancers are shown in Table 1. Classification by Dukes stage A–D was available for 81% of C18 cancers and for 69% of C19–21 cancers; by grade for 76% of C18 and 81% of C19–21 tumours; and by specified morphology for 86% of C18 and 92% of C19–C21. Tumours classed by the registry as ‘unknown’ for any of these variables include those recorded as ‘unstageable’ and those with data missing. Data completeness did not show material variation between men and women (Table 1) or by year of diagnosis; the proportion of tumours of both colon and rectum classed as stage unknown was higher in those over 75 years at diagnosis than in younger patients (∼25 *vs* ∼15%, respectively, for colon and ∼40 *vs* ∼25% for rectum). Age-standardised rates of registration of colorectal cancer were higher in men than in women and higher for colon than for rectal cancer throughout the period studied (Figure 1). Overall rates and distribution of cancers by stage remained relatively stable for the period 1995–2003 and particularly so for the 5 years 1999–2003 (Figure 2).

## DISCUSSION

Detailed population-based evaluation of routine registry data will be important for the accurate interpretation of changes in cancer trends following the introduction of screening for colorectal cancer. This study of data from one of the nine cancer registries in England found that information on stage, grade and morphology of tumours has been collected consistently for colorectal cancers registered in the past 10 years. Registration rates and distribution of cancers by stage, grade and morphology remained relatively stable for the period 1995–2003. Data on morphology were virtually complete for all cancers, but those on grade and stage were not. In particular, information on stage was not available for one-fifth of colon cancers and one-third of rectal cancers; and it was not always possible to distinguish between cancers recorded by the pathologist as ‘unstageable’ and those with missing data. This is a sufficiently high proportion of registered cancers to pose potential problems in the interpretation of future trends in stage registration in the context of the NHS Bowel Cancer Screening Programme. Stage data for colorectal cancer in the Oxford registry over the past decade have been consistently more complete than the average for UK Cancer Registries. Current quality control guidelines from the UK Association of Cancer Registries require only a minimum of 74% stage completeness for colorectal cancer (Department of Health, 2005) (a target higher than those for breast and cervical cancers; for many tumours, including those for which screening programmes may be considered in future, such as prostate cancer, there has been until very recently no required collection of stage data). Changes in cancer registration for colorectal cancer will be affected not only by true changes in the underlying incidence and stage distribution of cancers but also by changes in recording practices by pathologists and by cancer registries (Sharp, 2001). Some of these will be related to the screening programme itself; either directly, for example differences in data quality between screened and unscreened groups, or indirectly, for example earlier diagnosis owing to increased awareness of bowel cancer among doctors and among the public. During the next several years, there may also be changes in registration for all cancers as a result of increased and earlier ascertainment, of continuing improvements in quality of data collected by cancer registries and of the introduction of new data capture technology related to NHS Connecting for Health (http://www.icservices.nhs.uk/datasets/pages/cancer/). The current transition within cancer registration from use of Dukes stage to use of the TNM classification for recording stage of colorectal cancer will cause further problems. Some of these issues are illustrated in the present data: the rise in registration of Dukes stage A cancers seen between 1998 and 1999, and the corresponding fall in registrations with stage unknown, may reflect concurrent changes in pathology reporting, particularly of polyp tumours.

## Figures and Tables

**Table 1:** Cancers of the colon (ICD-10 C18) and of the rectum and related sites (ICD-10 C19-21) registered by Oxford Cancer Registry, 1995–2003

	**C18**	**Female C18**	**Male C18**	**C19-21**	**Female C19-21**	**Male C19-21**
Number of registrations	7865	3979	3886	4896	2076	2820
Mean age at diagnosis (years)	71.5	73.0	70.0	69.2	70.5	68.3
						
*Dukes stage*						
A	590 (8%)	286 (7%)	304 (8%)	686 (14%)	297 (14%)	389 (14%)
B	2386 (30%)	1206 (30%)	1180 (30%)	1019 (21%)	388 (19%)	631 (22%)
C	2097 (27%)	1042 (26%)	1055 (27%)	1177 (24%)	465 (22%)	712 (25%)
D	1323 (17%)	665 (17%)	658 (17%)	479 (10%)	190 (9%)	289 (10%)
Unknown	1469 (19%)	780 (20%)	689 (18%)	1535 (31%)	736 (35%)	799 (28%)
						
*Grade*						
Well differentiated	893 (11%)	435 (11%)	458 (12%)	634 (13%)	261 (13%)	373 (13%)
Moderately differentiated	4172 (53%)	2019 (51%)	2153 (55%)	2891 (59%)	1196 (58%)	1695 (60%)
Poorly differentiated	940 (12%)	511 (13%)	429 (11%)	451 (9%)	191 (9%)	260 (9%)
Undifferentiated	12 (<1%)	7 (<1%)	5 (<1%)	4 (<1%)	2 (<1%)	2 (<1%)
Unknown	1848 (24%)	1007 (25%)	841 (22%)	916 (19%)	426 (21%)	490 (17%)
						
*Morphology*						
Adenocarcinoma^a^	5978 (76%)	2915 (73%)	3063 (78%)	4014 (82%)	1630 (79%)	2384 (85%)
Mucinous adenocarcinoma^b^	779 (10%)	407 (10%)	372 (10%)	252 (5%)	106 (5%)	146 (5%)
Squamous cell carcinoma	0	0	0	177 (4%)	111 (5%)	66 (2%)
Other specified epithelial	0	0	0	48 (1%)	30 (1%)	18 (<1%)
Epithelial NOS	1089 (14%)	649 (16%)	440 (11%)	385 (8%)	186 (9%)	199 (7%)
Non-epithelial	7 (<1%)	3 (<1%)	4 (<1%)	12 (<1%)	9 (<1%)	3 (<1%)
Neoplasm NOS	12 (<1%)	5 (<1%)	7 (<1%)	8 (<1%)	4 (<1%)	4 (<1%)

a Includes two adenosquamous carcinomas.

b Includes signet ring carcinoma. NOS=not otherwise specified.
